# Community distribution of oxygen: a unique COVID-19 intervention

**DOI:** 10.1186/s41182-021-00333-z

**Published:** 2021-05-14

**Authors:** Nelson Ashinedu Ukor, Yusuff Adebayo Adebisi, Theogene Uwizeyimana, Attaullah Ahmadi, Osmond C. Ekwebelem, Precious Fadele, Don Eliseo Lucero-Prisno

**Affiliations:** 1 Faculty of Pharmacy, University of Port Harcourt, Choba, Nigeria; 2 Global Health Focus, London, UK; 3 African Young Leaders for Global Health, Abuja, Nigeria; 4 Department of Public Health, Mount Kenya University Rwanda, Kigali, Rwanda; 5 Medical Research Center, Kateb University, Kabul, Afghanistan; 6 Research and Development Hub, University of Nigeria, Nsukka, Nigeria; 7 Department of Medicine, University of Nigeria, Nsukka, Nigeria; 8 Department of Global Health and Development, London School of Hygiene and Tropical Medicine, London, UK

**Keywords:** COVID-19, Oxygen, Innovation, Community distribution, Telemedicine

## Abstract

The rapid spread of COVID-19 around the world has exposed some long-standing deficiencies in health systems, particularly in environments with low financial and medical resources. Most patients ill with COVID-19 require oxygen and supportive therapy for survival as there remains no conclusively established curative therapy. Following a number of critical research work and drawing from a millennia-long evolution of medical practice, respiratory support has been identified as a paramount intervention to ensure lives are saved when supportive care is required, and oxygen is an essential commodity to achieve this. This letter focuses on the numerous means for oxygen delivery to health facilities and in turn the end users and expands on the importance of innovation to improve oxygen supply. We describe a community distribution system with a telemedicine structure that can be leveraged for oxygen delivery.

To the Editor:

Following the designation of COVID-19 as a pandemic in early 2020, there have been consistent efforts to ensure that the best approach and standard of care is instituted in the patient management process. In managing COVID-19, like most other ailments associated with acute respiratory distress syndrome (ARDS), the prioritization of supportive care is crucial to patient survival. As such, interventions like respiratory support provide oxygenation and prevent progression to acute respiratory insufficiency which presents as a continuous challenge to critical care physicians [1]. Ensuring that patients who are critically ill with COVID-19 get adequate respiratory support remains a major challenge for various healthcare systems. However, previous experiences with ARDS have served to ease the knowledge gap regarding oxygen delivery strategies needed for its management, thereby setting the tone for future improvement [2].

Oxygen, being a major component of supportive care, needs to be prioritized to ensure more lives are saved since curative therapy for COVID-19 remains inconclusive. Evidence suggests strongly that oxygen is essential to the survival of patients with both acute and severe COVID-19 [3]. As such, ventilators cannot be put at the front burner of response because only a few patients may need them [4, 5]. For ventilators to be functional, oxygen must be readily available. Oxygen production should therefore be made a priority ahead of other measures. Important equipment like oxygen cylinders (filled from oxygen plants), oxygen concentrators (produced onsite from natural air), or liquid oxygen (piped oxygen systems) are required for the routine delivery of oxygen to patients. How these systems are employed hinges on human resource capacity and capability, the clinical and technical training protocols, local resources, and infrastructure available in the given setting [6]. In resource-poor settings, challenges to oxygen supply include inadequate financing, poor information systems, weak supply chain, poor human resource management, poor electricity supply and other infrastructural problems. Additionally, inadequate power supply poses critical challenge to the operation of oxygen plants and also threatens the smooth running of oxygen concentrators which require uninterrupted supply [7]. This has led to an increase in facilities with frequently empty or nonfunctional cylinders in low-resource settings [6].

With an increased uptake of testing, health systems are consistently challenged by the crowding of hospitals (tertiary care and other designated infection prevention and control centres) with many soon running out of space and operating beyond surge capacity. This strains the entire health care system and further assaults the already insufficient oxygen supply system [8]. Access to oxygen therapy is even further limited in low resource settings where a majority of patients requiring this life-saving therapy will not receive it. Focus should therefore shift from more complex structures like ventilator availability and intensive care capability to basic solutions that address the problem of oxygen shortage in these regions [5]. In responding to the dire challenges of a pandemic such as this, especially in areas where surge capacity is exceeded, making available makeshift community centres linked to major hospitals and home care will prove very beneficial and efficient in ensuring that transmission of infection is not escalated within the crowded facilities.

In patients with mild fluctuations/instability in oxygen saturation (SpO_2_), short-term oxygen therapy can be considered [9]. Using open wards (community centres or home-based settings), devices which can be readily moved for delivering oxygen (portable oxygen concentrators, compressed gas cylinders, portable continuous positive airway pressure (CPAP) machine, pulse oximeters) may prove beneficial [6] especially for patients that show normal compliance amidst hypoxemia (mild and moderate illness) as it ensures that these low priority patients have access to oxygen therapy. Provided oxygen administration can be done safely at home, this should be considered for such patients. However, if the patient remains clinically unstable or deteriorates, escalation to a specialized facility should be considered immediately.

The proposed innovation is for the selection of the most cost-effective option per context amongst available options such as: battery powered portable concentrators, portable lightweight cylinders [10], or rechargeable CPAP devices [11], and an appropriate delivery system (cannula, simple face mask, non-rebreathing mask, nose prong [10], double trunk mask [12], etc.). The selected device(s) can be easily transported from a central repository when the need arises. Each care facility will also be equipped with monitoring devices such as pulse oximeter, and thermometer. The aim of this intervention is to provide oxygen to mild to moderately ill individuals requiring short-term oxygen supply. This ensures that the already crowded dedicated treatment centres and hospitals are focused on handling severe cases. Consequently, expertise for maintenance and clinical decisions is confined at a central location that can be readily accessed by the surrounding primary care facilities in need of the services whether it is home care or a community centre.

To achieve an intervention such as this, developing the skills of health workers as well as primary caregivers and ensuring that teams (health workers, biomedical engineers, technicians, managers) work together is crucial. Telemedicine can be leveraged through the use of low bandwidth internet, short message service (SMS), and unstructured supplementary service data (USSD). Systems can be set up so that patients use direct phone calls, text messages, e-mails or mobile applications to send feedback to a central repository managed by adequately trained staff, usually a group of physicians trained in critical care and emergency medicine. Clinical information can as such be supplied by the patient or direct caregiver. Parameters that should be prioritized in facilitating the decision-making process for COVID-19 management will include: SpO_2_ (< 93%); heart rate (<45 beats/min or >120 beats/min); temperature (<35 °C or >38 °C); respiratory rate (<12 breaths/min or >20 breaths/min) [9].

To ensure efficiency of this system, a group of physicians and health care professionals with various specialties in critical care and emergency medicine should be responsible for determining patient progression and need for escalation rather than a standalone healthcare practitioner [9]. These professionals will be aggregated at the central point. By doing so, specialists are readily available to people in regions that would naturally be unreachable due to distance. Also, training of caregivers and family members primarily involved in direct patient care to identify and report clinical situation based on pre-defined parameters is important, as well as a proper understanding of the supply chain.

While device malfunction, lack of clinical expertise and rapid deterioration of patient health may threaten this system, continuous contact with supervising clinicians can help assuage these concerns. Other limitations to this system that may arise include the training required for makeshift health workers and primary caregivers (non-healthcare workers, family members) on infection prevention and control strategies, and identification of clinically significant parameters that require monitoring; high cost of transport for oxygen cylinder; and the specialization and expertise of transporters required. Network disruption in times of critical need and emergencies can also occur and should be considered when this intervention is to be implemented.

Concerns relating to access to the internet and mobile phone penetration may also rise. It is however noteworthy that more communities than ever before now have access to telecommunication devices. The Global Systems for Mobile Communications Association reported that commercial wireless signals cover over 85% of the world’s population as at 2009, reaching far beyond the electrical grid into hard-to-reach rural communities [13]. This notwithstanding, it is important to note that cost of adoption and other infrastructural problems leading to poor connectivity may still pose a barrier, in this case, service can be limited to only USSD and not internet broadband.

Depending on peculiar needs and resources available per setting, the following options can be considered: transport of oxygen in cylinders from centralized plant to point of care; use of transportable or portable oxygen concentrators to circumvent the need to transport oxygen over long distances in regions where constant electricity supply is available [14]. According to WHO, a total response to the supply of oxygen will mean that distribution systems are well placed with devices to deliver oxygen, pulse oximeters, ventilators where necessary, cylinders and sufficiently trained health workers are readily available [14]. This further emphasizes the need for disruptive innovations that ensures oxygen supply systems are not truncated and wider coverage is achieved, saving more lives in the process.

## Data Availability

Data sharing is not applicable to this article as no datasets were generated or analyzed.

## Abbreviations

COVID-19: Coronavirus disease
ARDS: Acute respiratory distress syndrome
ICU: Intensive care unit
PPE: Personal protective equipment
PaO_2_: Partial pressure of oxygen
SpO_2_: Oxygen saturation
WHO: World Health Organization
